# Effects of DCM Leaf Extract of *Gnidia glauca* (Fresen) on Locomotor Activity, Anxiety, and Exploration-Like Behaviors in High-Fat Diet-Induced Obese Rats

**DOI:** 10.1155/2019/7359235

**Published:** 2019-12-20

**Authors:** Wycliffe Makori Arika, Cromwell Mwiti Kibiti, Joan Murugi Njagi, Mathew Piero Ngugi

**Affiliations:** ^1^Department of Biochemistry, Microbiology and Biotechnology, School of Pure and Applied Sciences, Kenyatta University, P.O. Box 43844-00100, Nairobi, Kenya; ^2^Department of Pure and Applied Sciences, Technical University of Mombasa, P.O. Box 90420-80100, Mombasa, Kenya; ^3^Department of Environmental and Occupational Health, School of Environmental Sciences, Kenyatta University, P.O. Box 43844-00100, Nairobi, Kenya

## Abstract

Obesity is the main component of metabolic syndromes involving distinct etiologies that target different underlying behavioral and physiological functions within the brain structures and neuronal circuits. An alteration in the neuronal circuitry stemming from abdominal or central obesity stimulates a cascade of changes in neurochemical signaling that directly or indirectly mediate spontaneously emitted behaviors such as locomotor activity patterns, anxiety, and exploration. Pharmacological agents available for the treatment of neurologic disorders have been associated with limited potency and intolerable adverse effects. These have necessitated the upsurge in the utilization of herbal prescriptions due to their affordability and easy accessibility and are firmly embedded within wider belief systems of many people. *Gnidia glauca* has been used in the management of many ailments including obesity and associated symptomatic complications. However, its upsurge in use has not been accompanied by empirical determination of these folkloric claims. The present study, therefore, is aimed at determining the modulatory effects of dichloromethane leaf extract of *Gnidia glauca* on locomotor activity, exploration, and anxiety-like behaviors in high-fat diet-induced obese rats in an open-field arena. Obesity was experimentally induced by feeding the rats with prepared high-fat diet and water *ad libitum* for 6 weeks. The *in vivo* antiobesity effects were determined by oral administration of *G. glauca* at dosage levels of 200, 250, and 300 mg/kg body weight in high-fat diet-induced obese rats from the 6^th^ to 12^th^ week. Phytochemical analysis was done using gas chromatography linked to mass spectroscopy. Results indicated that *Gnidia glauca* showed anxiolytic effects and significantly increased spontaneous locomotor activity and exploration-like behaviors in HFD-induced obese rats. The plant extract also contained phytocompounds that have been associated with amelioration of the main neurodegenerative mediators, viz., inflammation and oxidative stress. These findings provide “qualified leads” for the synthesis of new alternative therapeutic agents for the management of neurologic disorders. However, there is a need to conduct toxicity studies of *Gnidia glauca* to establish its safety profiles.

## 1. Introduction

Metabolic abnormalities stemming from abdominal or central obesity are increasingly linked to impairments in central nervous system (CNS) function [1]. The hypertrophied and hyperplastic adipose tissue stimulates a cascade of changes in neurochemical signaling that directly or indirectly mediate behaviors [2, 3]. Ostensibly, this degree of relative adiposity is implicated in a wide range of neurobehaviors among which are spontaneously emitted behaviors (activity patterns, anxiety, and exploration), motivated behaviors (feeding, drinking, sexual behavior), and operant performance, attentional processes, learning, and memory [4, 5].

The obese phenotype and/or chronic exposures to a high-fat diet (HFD) markedly exacerbate the odds of developing spontaneously emitted behaviors [6]. A study indicated that mice fed on HFD for 12 weeks showed reduced locomotor and exploratory behaviors in open-field tests and elevated plus maze as well as depressive-like features characterized by reduced ambulatory activity in the forced swim tasks [5].

Exploration is one of the main domains of behaviors referring to the tendency to investigate a novel environment [7]. It is closely related to curiosity [7]. The cognitive map theory postulates novelty as a misrepresentation of an item or place in the cognitive mapping/locale system [8]. The locale system is located within the hippocampus containing mental representations of previously perceived stimuli. Therefore, the hippocampal system supposedly signals a lack of information about the current environment and exploration becomes a direct response to the mismatch detected [8].

Chronic exposure to obesogenic diets is often associated with physical inactivity due to altered coordination of motor and reflexive responses [9, 10]. Increased adiposity alters motor function through enhanced decrements in balance, muscle strength, and coordination [11]. Deficits in motor performance might be due to alterations in the striatal dopaminergic signaling or in the cerebellum [12, 13]. Deficiencies in dopamine synthesis, striatal dopamine release, and defective striatal dopamine receptors are associated with impairments in striatal dopamine function [14, 15].

Anxiety disorders, being the most prevalent mental disorders, globally contribute to reduced quality of life and predispose affected individuals to other psychiatric comorbidities [16, 17]. Anxiety, fear, chronic worry, muscle tension, panic attacks, and apprehension are the main psychological symptoms while physical symptoms involve chest dysphoria, fatigue, and tension [18]. Anxiety disorders are majorly categorized into specific and social phobias, panic disorder, obsessive-compulsive disorder, posttraumatic stress disorder, and generalized anxiety disorder [18].

Studies have reported a positive association between obesity and anxiety disorders such as panic disorder, specific phobia, and social phobia [19–21]. Obesity as a causal factor for anxiety involves several paths such as social discrimination against obese persons [22], low self-esteem in an unfriendly social network [23], distress from illness burden (especially diabetes mellitus, asthma, and cardiovascular diseases), and adverse drug effects [24]. Anxiety disorders as the causal factor for obesity have been correlated with disruption of hypothalamic-pituitary-adrenal (HPA) axis which results in dysregulation of autonomic functions [25, 26]. These factors create another vicious cycle (obesity-anxiety cycle).

Inflammation and oxidative stress due to increased adiposity play a pivotal role in the pathogenesis of neuropsychiatric disorders through their effect on the hypothalamus, amygdala, and the hippocampus [27]. Chronic consumption of high-fat diet stimulates production of proinflammatory cytokines (TNF-*α*) [28], interleukins (1*β*, 2, 6, 8, and 12) (Sahebkar, 2014), chemokines, immune cells, prostaglandins, and nitric oxide which in turn precipitate hypothalamic-mediated oxidative stress [29]. The compromised redox homeostatic status characterized by attenuated antioxidant defenses activates the brain's resident immune cells, the microglia, and astrocytes, to further produce inflammatory mediators, which exacerbate oxidative damage in the hippocampus [30].

Pharmacological agents available for the treatment of neurologic and psychiatric disorders have had limited potency or intolerable adverse effects [31]. Therapeutic herbs and nutrients have, therefore, provided an effective alternative treatment with minimized side effects and capacity to potentiate the effects of prescribed medications [32]. The present study is aimed at determining the modulatory effects of DCM leaf extract of *Gnidia glauca* on locomotor activity, exploration, and anxiety-like behaviors in HFD-induced obese rats in an open-field arena. The generated data will provide “qualified” leads in drug design from this plant for the treatment and/or management of neurologic disorders.

## 2. Materials and Methods

### 2.1. Processing and Extraction of the Plant Material

Fresh leaves of *G. glauca* were dried under a shade at room temperature for 21 days. By the use of an electric mill, the dried leaves of *G. glauca* were ground into a fine powder. The milled plant sample was then kept at room temperature free from direct sunlight in a dry airtight plastic container prior to extraction. In one liter of dichloromethane (DCM), 500 grams of the powdered sample of *G. glauca* was added and soaked for forty-eight hours. The dissolved compounds were decanted and filtered using muslin cloth into a dry clean conical flask. The filtrate was concentrated under reduced pressure by use of a rotary evaporator at 40°C to attain a semisolid residue [33]. The yield of the plant extract was determined and subsequently refrigerated at -20°C prior to its analysis.

### 2.2. Obesity Induction

Obesity was induced by feeding the experimental animals with a high-fat diet and regular supply of water for twelve weeks. The ingredient of the experimental diet was done according to a formula as described by Srinivasan et al. [34] with minimal moderation as indicated in Table 1.

The obesity index was defined by Lee index. The Lee index was calculated according to the formula described by Lee [35].

(1) $$\begin{array}{c} \text{Lee index }\left(\%\right)=\sqrt[{3}]{\frac{\text{ Body weight }\left(\text{g}\right)}{\text{Nose to anus length }\left(\text{cm}\right)}}\times 100. \end{array}$$

Rats with Lee obesity index value (equivalent to BMI ≥ 30 in humans) of 310 and above were considered obese [35] and used in the study. Following exposure to HFD (except for normal control group) for 6 weeks, all the rats in the negative control, positive control, and extract-administered experimental groups attained the target diagnostic value of obesity, indicating the end to the obesity induction phase. The naso-anal lengths (NAL) (cm) of rats were measured by a nonextensible thread and readings taken using a ruler with an accuracy of 0.1 cm.

### 2.3. Experimental Design

Thirty female rats were randomly grouped into 6 different sets of 5 animals each. The normal control group (Group I) comprised of normal animals that were fed on a standard chow diet for 12 weeks. Besides, no treatment was given to this group of animals. Group II (negative control) comprised of rats that were fed on HFD for 12 weeks to induce obesity. The positive control group (Group III) consisted of HFD-induced obese animals that were administered with the reference drug, orlistat, from the 6^th^ to 12^th^ week. Group IV-VI (experimental groups) comprised of HFD-induced obese rats that were administered with the DCM leaf extract of *G. glauca* at different doses of 200, 250, and 300 mg/kg body weight from the 6^th^ to 12^th^ week. All the treated rats were maintained on a high-fat diet throughout the dosing period. Further, all the experimental rats received water *ad libitum* in the entire study period.

### 2.4. Open-Field Arena

In order to determine whether *G. glauca* modulates gross locomotor activity, exploration, and anxiety-like behaviors in HFD-induced obese rats, animals were tested in an open-field arena [36] after 6 weeks of oral administration of therapeutic doses of the plant extract.

#### 2.4.1. Apparatus

**Figure 1:**
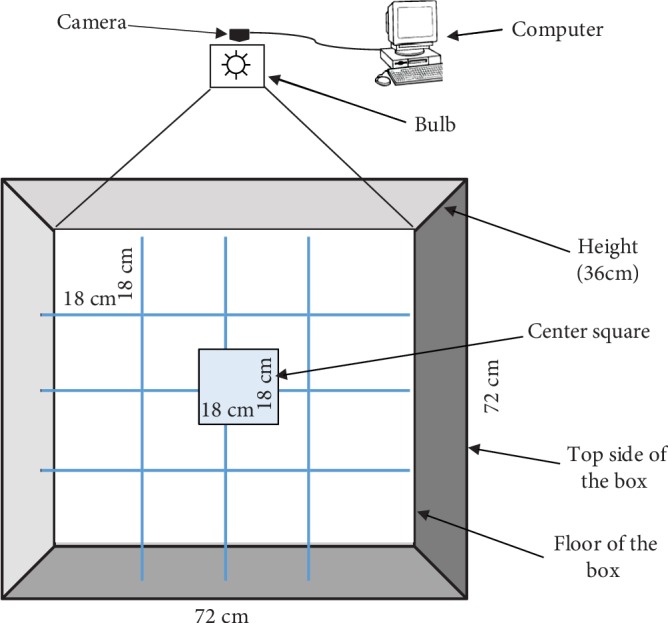
The open-field arena.

The open-field apparatus consisted of an open-top box (72 cm × 72 cm) with 36 cm high walls. Blue visible lines were drawn on the floor using a marker into sixteen 18 × 18 cm squares (Figure 1). A center square (18 cm × 18 cm) was drawn in the middle of the arena (within the four inner squares) with a red marker (Figure 1). A 60-Watt white light bulb provided lighting. The floor was covered with a sheet of clear Plexiglas which was cleaned using 70% ethyl alcohol after testing each animal. Animal behaviors in the arena were recorded and tracked by an overhead video camera connected to a PC with EthoVision XT software (Figure 1).

#### 2.4.2. Experimental Procedure

The animals were transferred to the testing room in their home cages and allowed to acclimatize to this room prior to testing. Each rat was gently placed in the center of the open-field arena and left freely to explore the arena for 5 minutes while recording scores of its behaviors. At the end of the 5-minute test period, animals were returned to their respective home cages. The Plexiglas was removed, cleaned, and disinfected with 70% ethyl alcohol after each trial.

To determine the effect of the DCM leaf extract of *G. glauca* on locomotor activity, exploration, and anxiety-like behaviors, the following animal behaviors were assessed: number of line/grid crossing (frequency with which the rats crossed one of the grid lines with all four paws), center square entries (frequency with which the rats crossed one of the red lines with all four paws into the central square), latency period (duration of stay in the central square), rearing (frequency with which the rats stood on their hind legs in the arena), stretch attend postures (frequency with which the rats demonstrated forward elongation of the head and shoulders followed by retraction to their original position), grooming (duration of time the animal spent licking or scratching itself while stationary), freezing (duration with which the animal was completely stationary), urination (number of puddles or streaks of urine), and defecation (number of fecal boli produced per animal) [36].

Locomotor activity for each animal was assessed by the sum of line/grid crosses and the number of rears. The behavioral domains used to test exploration-like behaviors included the frequency of rearing, central square entries, and latency in the central square. The animal behavioral domains used to assess anxiety were latency period, fecal boli score, frequency of urination, grooming, stretch attend postures, and freezing.

### 2.5. Gas Chromatography-Mass Spectrometry Analysis

Sample analysis was determined using GC-MS (7890/5975 Agilent Technologies, Inc., Beijing, China) consisting of a gas chromatograph integrated into a mass spectrometer instrument. The GC-MS was equipped with a HP-5 MS (5% phenyl methyl siloxane) low bleed capillary column of 0.25 *μ*m film thickness, 0.25 mm diameter, and length of 30 m. An electron ionization system with ionization energy of 70Ev was used in GC-MS detection. Helium (99.99%) gas carrier was used at a consistent flow rate (1.25 ml/min) in split mode. The mass transfer line and injector temperature were set at 200°C and 250°C, respectively. One microliter was used as an injection volume. Oven temperature was programmed from thirty-five degrees Celsius for five minutes, with an elevation of ten degrees Celsius per minute to two hundred and eighty degrees Celsius for 10.5 minutes, followed by fifty degrees Celsius per minute to two hundred and eighty-five degrees Celsius for 29.9 minutes with seventy minutes run time. The mass spectrometry operating parameters included ionization energy, 70 eV; ion source temperature, 230°C; relative detector gain mode, scan speed 1666 *μ*/sec; solvent cut time, 3.3 min; the interface temperature was 250°C, scan range 40-550 m/z.

### 2.6. Data Management and Statistical Analysis

To assess the performance in the open-field arena, each determinant of the behavioral domain was recorded and tracked by an overhead video camera connected to a PC with EthoVision XT software. The data for each behavioral domain was exported to Microsoft® Excel spreadsheet, where it was organized and later transferred to statistical software Minitab (Version 17.1) for analysis. The data was found to conform to the assumptions of parametric data. One-way ANOVA was used to test the significant differences among the normal control group rats, negative control group rats, orlistat-treated group of rats, and an extract-treated groups of rats. The data was further subjected to Tukey's post hoc for pairwise separation and comparison of means. The criterion for significance was set at *p* ≤ 0.01. The findings were presented in a table.

## 3. Results

### 3.1. Effect of DCM Leaf Extract of *Gnidia glauca* on Locomotor Activities, Anxiety, and Exploration-Like Behaviors in HFD-Induced Obese Laboratory Rats

Treatment of HFD-induced obese rats with DCM leaf extract of *G. glauca* resulted in a significantly higher grid crossing score relative to the negative control group rats (*p* ≤ 0.01; Table 2). Further, rats treated with the plant extract showed a higher grid crossing than those in the normal control group (*p* ≤ 0.01). Administration of extract dosages of 250 and 300 mg/kg body weight resulted in a significant increase in grid crossings than those of rats treated with the reference drug, orlistat (*p* ≤ 0.01; Table 2).

It was further observed that HFD-induced obese rats treated with the plant extract had a higher number of rearing episodes relative to HFD-fed untreated rats (Table 2). Besides, there was no significant variation in the number of rearing episodes observed among extract-treated rats at dosages of 200 and 250 mg/kg body weight and those of normal control group rats and rats in the positive control groups (*p* > 0.01). Results indicated higher defecation and urinating episodes in rats in the negative control group than those of rats in the extract-treated groups (*p* ≤ 0.01). Similarly, the number of stretch attend postures and urinating episodes was significantly high in HFD-fed untreated rats relative to the extract-treated group of rats (*p* ≤ 0.01). Further, it was observed that the number of visits to the central square after the initial exit was more on those groups of rats that were treated with the plant extract and the reference drug, orlistat, than HFD-fed untreated obese rats (Table 2). Remarkably, no significant difference in defecation, urination, center square entries, and stretch attend postures was observed among rats in the extract-treated, positive control, and normal control groups (*p* > 0.01; Table 2).

The results also showed that the HFD-fed untreated obese rats in the negative control group had a significantly longer latency period in the central square upon entry into the open-field arena than rats treated with *G. glauca* leaf extract (*p* ≤ 0.01). Treatment of rats at dosage levels of 250 and 300 mg/kg bodyweight of the extract resulted in a shorter latency period in the central square than rats administered with the reference drug, orlistat (Table 2). However, the latency period was statistically similar in rats treated with the reference drug, orlistat, and those in the normal control group (*p* > 0.01).

The results also revealed that rats in the negative control group substantially froze longer upon exposure to the open-field arena than rats treated with the three extract doses (Table 2). However, the effect was not statistically significant among rats treated with the reference drug, orlistat, and those treated with the plant extract at dosage levels of 200 and 250 mg/kg body weight (*p* > 0.01). Treatment of rats with the highest extract dose of 300 mg/kg body weight significantly reduced immobility time (freezing period) than orlistat-treated rats (*p* ≤ 0.01; Table 2).

Results also demonstrated that the administration of *G. glauca* leaf extract significantly reduced the grooming behavior in HFD-induced obese rats relative to untreated obese rats in the negative control group (*p* ≤ 0.01). However, the propensity to groom in the extract-treated groups of rats and those treated with the reference drug, orlistat, were comparable (*p* > 0.01; Table 2).

### 3.2. The Concentrations of Compounds Identified in DCM Leaf Extract of *Gnidia glauca*

The gas chromatography-mass spectrometry analysis of DCM leaf extract of *G. glauca* indicated the presence of oleic acid, *γ*-sitosterol, curcumin, quercetin, stilbenes, phytol, octadecanoic acid (stearic acid), gallocatechin-catechin flavan, ferulic acid, flavonols, and others (Table 3).

## 4. Discussion

The open-field test provides simultaneous measures of locomotion (ambulatory activity), exploration, and anxiety (emotionality) [37]. It is a model not only useful in the assessment of the behavioral performance of the test animals but also contributes knowledge on the neurobiological mechanisms mediating behaviors [37].

Locomotor/ambulatory activity is the function of performance on motor tasks while exploration or novelty may involve some quality never previously experienced or familiar items arranged in unfamiliar ways [38]. Exploratory behavior is thus curiosity and attraction to novelty [38]. Animal behaviors such as frequency of line crosses, frequency of rearing, central square entries, and latency in the central square are used as measures of locomotor activity and exploration. A higher frequency of these parameters indicates increased locomotion and exploration and vice versa [37]. The present study indicated a number of clear effects on these behavioral domains upon treatment with the DCM leaf extract of *G. glauca*.

The frequency of line/grid crosses measures the horizontal exploration or locomotor behavior and represents the horizontal distance covered [38]. Line crossing is the frequency with which the rat crosses each of the lines with all four paws. In the present study, line crossing was significantly (*p* ≤ 0.01) increased in the extract-treated rats as compared to the HFD-fed untreated obese rats. Similar studies indicated that the administration of ethanolic extract and acetone extract of *Cedrus deodara* increased locomotor activity in neonatal rats [39]. In normal circumstances, rats naturally move in order to find the location of feeds, gather nesting materials, search for nesting places and sexual partners, or flee themselves from enemies [40]. However, the reduced locomotor activity observed in HFD-fed untreated obese rats could be due to the overweight nature of the animals, generalized muscle fatigue, and/or increased behavioral despair [38]. Besides, the diminished ambulatory/locomotor activity could be a result of the damage in the primary motor area and/or distress from the illness burden associated with increased adiposity [25, 38]. The ability of the extract to reduce body weight might be attributed to the observed increased ambulatory activity in extract-treated rats. Moreover, the extract might have resulted in a positive effect on striatal dopaminergic signaling through increased striatal receptor sensitivity and dopamine synthesis thereby improving motor activity [13].

The frequency of rearing or vertical exploration was significantly decreased in HFD-fed untreated obese rats as compared to their treated counterparts (*p* ≤ 0.01). Rearing measures exploratory behavior or otherwise vertical locomotor activity. When rearing, the animal stands upright on its hind limbs often using their tail as support with its forelimbs freely suspended in the air or resting on the wall of the open-field arena. Through rearing, olfactory signals can be taken in from the air, as well as visual cues [41]. Locomotor activity is driven by exploration since its reduced form could reflect reduced exploration as it is accompanied by reduced rearing frequency [41]. Consistent with this study, it was demonstrated that oral administration of ethanolic extracts of *Nauclea latifolia* and *Emilia sonchifolia* increased locomotion and exploratory activities as evidenced with a high frequency of rearing in mice [38].

The state of being obese is associated with decreases in the motor output, often termed as “physical inactivity” [10]. Chronic exposures to obesogenic diet contribute to striatum damage thereby affecting the dopamine synthesis and release as well as striatal-dopamine receptor function [13]. The striatal-dopamine plays a key role in the proper control of movement, and therefore, its impairment contributes to physical inactivity in obesity akin to classical movement disorders such as Parkinson's disease [13, 42]. The motivated locomotor and exploratory behaviors observed in extract-treated rats could been linked to facilitated dopamine synthesis, release, and restoration of striatal-dopamine receptor function [43, 44].

The chronic mobility problems of joints and muscles in obese patients are largely contributed by alteration of motor circuitry in the brain [13]. Besides, the obesity-induced adaptations due to altered motor circuitry could continue to contribute to physical inactivity even after weight loss [13]. The reduction of expression levels of brain-derived neurotrophic factor (BDNF) and its tyrosine kinase receptor, TrkB, in hypothalamic nuclei affects the strength of synaptic connections or dendritic spine density leading to altered satiety signals and locomotor activity [45]. High-fat diets potentiate an oxidative attack on brain resident cells resulting in activation of cholinergic motor inhibitory system [46]. Alteration of the activity of acetylcholinesterase (AchE) and damage to the peripheral muscle due to necrosis of skeletal muscle fibers enhances the reduction of locomotor activity in animal models [47].

The increased ambulatory or spontaneous physical activity (SPA) characteristic to extract-treated rats could also be as a result of the action of neuropeptide, orexin A, independent of feeding behavior [48]. Orexin A robustly stimulates spontaneous physical activity and nonexercise activity thermogenesis [49, 50]. Central administration of orexin-A (into the hypothalamic paraventricular nucleus) was found to increase rearing frequency and locomotor activity in rats [50]. The orexin neurons project to the dopaminergic neurons in the substantia nigra that innervate the striatum and forms a critical component of motor activity [51]. Therefore, an alteration in the expression of orexin, and/or its signaling, could exacerbate spontaneous physical inactivity and contribute to weight gain [51].

The test for anxiety is usually based on the conflicting tendencies of rats to explore a novel environment in contrast to the aversive features of a brightly lit open arena or an elevated space [37]. Moreover, in the open-field arena, this behavioral domain (anxiety) may be mediated by two key factors, namely, agoraphobia and individual testing. Agoraphobia is a function for anxiety based on the size of the test area relative to the size of an animal while individual testing is a function for anxiety based on the separation of an animal from its social group [52]. Animal behaviors such as increased latency period, greater fecal boli score, higher frequency of urination, increased grooming period, fewer rears, higher frequency of stretch attend postures, and increased freezing duration are used as measures of anxiety. A higher frequency or an increased duration of these parameters indicates increased anxiety [37].

Analysis of stretch attend postures (SAP) revealed a significantly increased frequency in obese untreated rats relative to extract-treated rats (*p* ≤ 0.01). The SAP is the frequency with which the animal demonstrated forward elongation of head and shoulder followed by retraction to its original position. These are risk assessment behaviors of fear and anxiety which indicates that the animal is hesitant to move from its present position of comfort to a new position. Thus, decreased levels of this behavior are indicative of a low level of anxiety and fear and vice versa [53]. These results were consistent with earlier findings that mice with HFD-induced obesity demonstrated a high frequency of SAP relative to obese mice treated with the herbal extracts from *Morus alba*, *Melissa officinalis*, and *Artemisia capillaris* [54].

Results also showed that the extract-treated rats had an increased frequency of entry to the inner zone of the open-field arena relative to HFD-fed untreated obese rats (*p* ≤ 0.01). A high frequency of movements into the center of the arena in open-field tests is reflective of reduced anxiety, increased locomotor activity, and exploration [37]. Rats are generally thigmotactic; they avoid open areas and prefer moving alongside walls where they perceive tactile stimuli via their vibrissae [41]. However, when the animal is less anxious, their exploratory behavior increases and tends to move all over the holding cage or arena. Consistent with the present study, previous studies observed that the treatment of mice with hydroalcoholic extract of *Coriandrum sativum* increased the frequency of entry to the inner zone of the open-field arena [55]. The sedative and muscle relaxant effects of *Coriandrum sativum* are indicative of its anxiolytic effects [55].

The HFD-fed untreated group showed longer latency period in the central square upon entry into the open-field arena, an indicator of higher anxious states due to anxiogenic effects of chronic exposure to high-fat diets. The quicker the retreat from the center square of the arena in extract-treated rats is indicative of increased automatic and exploratory behaviors due to extracts anxiolytic effects [56]. These findings were in agreement with another study that demonstrated that HFD-induced obese rats showed less explorative interest due to fewer cross-lattice numbers and reduced percent of time spent in the center of the arena and open arms [57]. The reduction in explorative interest in HFD-induced obese untreated rats appears to be symptoms of depressive disorders consistent with those observed in patients suffering from anxiety disorders [58]. Current studies have demonstrated that chronic intake of HFD has led to depressive- and anxious-like behaviors [57, 59].

Immobility time (freezing duration) was significantly (*p* ≤ 0.01) increased in HFD-fed untreated rats compared to extract-treated rats, an indicator of increased anxious state and hypoactivity or impaired locomotor activity [37]. Freezing often occurs in response to a sudden change in the surroundings where the animal usually stands still with its forelegs raised while looking up. Previous studies demonstrated that HFD-induced obese rats exhibited a significantly low frequency of rearing as compared to rats treated with therapeutic doses of aqueous extract of *Ginkgo biloba* [60]. The anxiolytic effects of the *G. glauca* leaf extract might be accompanied by increases in the brain levels of monoamines such as serotonin, norepinephrine, and dopamine [61]. Serotonin and norepinephrine are neurotransmitters that play a key role in mood regulation [62, 63].

Increased fecal boli and urination scores observed in HFD-fed untreated obese rats are suggestive of fear and anxious states. Comparatively, the low scores of these parameters in the extract-treated rats may be attributed to the presence of bioactive chemicals responsible for downregulation of receptors and connectivity in the amygdala, a key center of fear [25, 37]. Previous studies have demonstrated that high-fat feeding and obesity increase the production of BDNF and phospho-CREB in the striatum contributing to negative emotional states and depressive-like symptoms [5]. This biochemical alteration in the brain reward circuitry could be implicated for the observed increased fecal boli and urination score in HFD-fed untreated obese rats.

Grooming is a dearousing self-directed behavior associated with anxiety upon displacement of animals into a novel environment or aversive situations such as an open-field arena [64]. Grooming duration is the time the animal spends licking or scratching itself with paws and face washing actions while in a stationary position. This stereotypical behavioral sequence is usually increased in anxious states. Anxiolytic drugs, however, reduce the grooming behavior, whereas anxiogenic drugs facilitate grooming [41]. Treatment with therapeutic doses of the plant extract led to a significant decrease in grooming duration relative to HFD-fed untreated obese rats (*p* ≤ 0.01). Consistent with this study, the open-field tests performed to assess the neurobehavioral effects of *Nauclea latifolia* and *Emilia sonchifolia* [38] and *Mammea africana* [65] in rodents indicated reduced grooming frequencies together with increased spontaneous locomotion and exploratory activities [66].

Self-grooming is a highly stereotyped pattern of sequential movements (syntactic chain pattern) that is modulated by circuits that incorporate the basal ganglia such as the striatum, substantia nigra, and nucleus accumbens in the forebrain [64, 67]. Striatal circuits majorly subserve the basal ganglia and are mainly involved in learning, motivation, and motor sequencing [68]. Lesions of the striatum completely impair sequential syntactic self-grooming chains [64]. The limbic circuitry that includes the amygdala and the hypothalamus also modulates self-grooming behavior in rodents. The amygdala mainly modulates motivational states, such as desire, fear, and anxiety [69]. Studies showed correlations between reduced dopamine release within the amygdala and increased anxiety-like behavior in low- and high-grooming rats, respectively [70].

The hypothalamic paraventricular nucleus is another limbic region that has been implicated in the regulation of self-grooming in rodents [70]. Besides, the hypothalamic-pituitary stress-related hormones such as corticotropin-releasing hormone (CRH) and adrenocorticotropic hormone (ACTH) also influence self-grooming in rodents [64]. The anxiolytic effects observed in the extract-treated rats as evidenced by reduced self-grooming increased exploratory and locomotor activities and could be attributed to the extracts' effect on the dopamine release in the nigrostriatal and mesolimbic systems [71]. Dopamine plays a critical role in locomotor function, self-grooming, and other complex behavioral patterns [64]. The *G. glauca* leaf extract might have also contributed to a reduction of stress-induced self-grooming by enhancing the GABAergic tone through attenuating the intensity of the perception of anxiogenic stimuli [71, 72].

The mechanistic basis underlying obesity as a causal factor for anxiety could relate to oxidative stress and inflammation [30]. Studies have demonstrated that exposures to chronically high-energy diets influence the activity of glial cells that mediates endogenous immune system within the microenvironment in the CNS [73]. The activation of glial cells is the hallmark of inflammation in the brain [74]. Activated microglia produce neurotoxic inflammatory stress signals, such as tumor necrosis factor-alpha (TNF-*α*) [28], interleukins (1*β*, IL-2, IL-6, IL-8, and IL-12) [75], lipoxygenase [76], cyclooxygenase-2 (COX-2) [77], monocyte chemoattractant protein (MCP) [26], growth factors, and complement proteins [78]. These proinflammatory mediators, in turn, precipitate an inflammatory signaling cascade by activating other resident cells to produce additional molecules that perpetuate microglia activation in a positive feedback loop [73].

Increased inflammation due to chronic exposure to high-fat diet increases the vulnerability of neurotransmitter receptors to oxidative stress through activation of the oxidative stress-sensitive nuclear factor-kappa-*β* (NF-*κβ*) [79]. The activated NF-*κβ*, in turn, upregulates the inflammatory response resulting in a further increase in ROS such as superoxide species and nitric oxide (NO) as well as increased expression of inducible nitric oxide synthase (iNOS) [80]. High levels of ROS exacerbate oxidative stress and inflammation, and thus, vulnerability to further stressors [81]. Facilitated central adiposity might also precipitate oxidative damage due to compromised redox homeostatic status characterized by attenuated antioxidant defenses thus exacerbating a neuropsychiatric damage [30, 82]. The therapeutic effects exhibited by the *G. glauca* leaf extract could be due to its ability to mitigate inflammation and oxidative stress by downregulating the activity and release of proinflammatory mediators and restoration of redox homeostatic status through activation of antioxidant defenses [26]. The normalization of NF-*κβ* levels reduces the expression of the proinflammatory cytokines and consequently results in low levels of ROS in the hippocampus [83].

Pharmacological manipulations of anxiety disorder by anxiolytic agents like benzodiazepines (BDZs) and allopregnanolone enhance GABAergic tone. Binding of the anxiolytic agents to one of the two gamma subunits of the GABA-A receptor causes a structural modification of the receptor and allosterically increases GABA-A receptor activity [84, 85]. This binding also facilitates the opening of GABA-activated chloride channels thereby increasing chloride ion conductance and inhibition of the action potential [86]. The eventual allosteric binding of GABA to the gamma subunit of the GABA-A receptor decreases the excitability of neurons and augments a calming effect [85, 86]. The observed anxiolytic properties of the *G. glauca* leaf extract may be attributed to stimulation of the binding of gamma-aminobutyric acid (GABA) to GABA-A receptors that occurs abundantly on the surfaces of neurons in the amygdala and other parts of the limbic system and, therefore, results in a calming effect [25].

In the present study, the observed anxiolytic effects and increased locomotor and exploration-like behaviors in extract-treated rats could be attributed to the presence of some bioactive compounds in the DCM leaf extract of *G. glauca*. The synergistic and/or additive effects of these phytochemical compounds might be implicated in amelioration of symptomatic complications of obesity, viz., anxiety, locomotor activity, and exploration.

The phenolic compounds such as catechins and epicatechins were found to confer neuroprotective effects by mitigating oxidative and metabolic insults [87]. Catechins exhibit neuroprotective activities by activating multiple signaling pathways that exert cell survival and anti-inflammatory actions, including altering the expression of proapoptotic and antiapoptotic proteins and upregulating antioxidant defenses [88]. Catechins activate protein kinase C (PKC) and transcription factors that promote the expression of cell survival genes [89]. Studies reported that catechins and epicatechins exhibited protective effects of dopaminergic neurons from damage induced by 6-hydroxydopamine in a rat model of Parkinson's disease [87]. In addition, catechin and epicatechin suppress neuroinflammation, attenuate activation of microglia, and inhibit the release of the mediators associated with the apoptotic death of neurons [90].

Curcumin was shown to ameliorate impaired hippocampal neurogenesis and increase the expression levels of brain-derived neurotrophic factor (BDNF) in severely stressed rats [62]. Curcumin has been used in the prevention and management of neurodegenerative diseases such as Alzheimer's disease, Parkinson's disease, and stroke [62]. It mitigates oxidative stress and inflammation by downregulating the activity of lipoxygenase, COX-2, and inhibiting the generation of proinflammatory cytokines such as TNF-*α*, interleukins I, II, VI, VIII, and XII, and monocyte chemoattractant protein (MCP) [75, 91]. Besides, curcumin deactivates the transcription factor NF-*κβ* through induction of the expression of antioxidant enzymes and Bcl-2 [92]. Curcumin activates multiple signaling pathways through ligand binding to various receptors that include growth factor receptors (GFR), G protein-coupled receptors (GPCR), and insulin receptors (IR). These receptors, in turn, activate the kinase cascades involving phosphatidylinositol-3-kinase (PI3K), mitogen-activated protein kinases (MAPK), and protein kinase C (PKC) [92].

Quercetin has been shown to improve brain cell function and signaling by mitigating extraneuronal parameters of survival—the oxidative stress [93]. Quercetin reduced oxidative stress and protected cultured hippocampal neurons against nitric oxide-mediated cell death [87]. Quercetin ameliorates calcium dysregulation thereby protecting from ischemic injury, neuronal cell death, and consequent brain damage [94]. Quercetin treatment decreased acid-mediated intracellular calcium levels and inhibited spectrin breakdown by inactivation of calcium-dependent protease cabin [95]. Quercetin significantly decreased protein oxidation, A*β*-induced toxicity, and apoptosis in primary hippocampal cell cultures [94]. This novel antioxidant offers an effective and safe means of bolstering the body's defense against free radicals [96].

Stilbenes such as pinosylvin and resveratrol are phytophenols that have been shown to exhibit antioxidant activity [97]. Pinosylvin and resveratrol protected cultured neurons against oxidative damage by scavenging nitric oxide radicals [42]. Similarly, in a model relevant to Parkinson's disease, resveratrol protected cultured dopaminergic neurons against oxidative-induced cell death [42]. Current findings indicate that the administration of resveratrol and/or pinosylvin to rats confers the protection of neurons in the brain and spinal cord from ischemic injury [42]. In models relevant to Alzheimer's disease, stilbenes promoted clearance of amyloid *β*-peptide from cultured cells hence preventing neuronal cell damage [98].

Flavonoids were reported to modulate neuronal function and prevent neurodegeneration [99]. Flavonoids were shown to improve memory and learning through stimulation of neuronal regeneration and enhancement of neuronal function [92]. They inhibit TNF-*α*, IL-1*β*, and nitric oxide in activated microglia cells [92]. Flavanone maintains nigrostriatal integrity and functionality and serves as a potential neuroprotective agent against 6-hydroxydopamine [99]. Flavonoids activate the P13-kinase-mTOR cascade and ERE-CREB pathway resulting in changes in synaptic plasticity. Flavanones were found to inhibit oxidative-induced neuronal apoptosis through phosphorylation of signaling proteins essential in prosurvival pathways. Neryl acetate has been observed to activate specific Ca^2+^ channels by its action on vanilloid receptors [92].

Previous studies reported that condensed tannins such as gallocatechin-catechin flavan and anthocyanins can diffuse through the central nervous system and cross the blood-brain barrier (BBB) [100]. Gallocatechin-catechin flavan and anthocyanins confer neuroprotective function through their antioxidative properties. In human SH-SY5Y neuroblastoma cells, condensed tannins reduced A*β*-induced neurotoxicity by enhancing the formation of A*β* fibril formation thus reciprocally modulating local A*β* clearance [101]. Gallocatechin-catechin flavan and anthocyanins have been shown to have potent anti-inflammatory activities. They inhibit inflammatory mediators COX-2 [102].

Alkaloids increase gamma-aminobutyric acid (GABA) in the synapses of the brain [103]. They are highly potent vasodilator agents, enhance cerebral blood flow, facilitate glucose uptake by brain cells, and protect from hypoxia and ischemia [103].

Ellison and *α*-amyrin were shown to activate the transient receptor potential (TRP) ion channels in the cell membrane of neurons [104]. This resulted in Ca^2+^ influx which in turn activates neuroprotective kinase signaling cascades via cAMP response element binding protein (CREB) and mitogen-activated protein kinases [104]. The CREB stimulates expression levels of a major neurotrophic factor, the brain-derived neurotrophic factor (BDNF). The BDNF activates the PI-3K/Akt and MAPK/ERK pathways through binding to its tyrosine kinase TrkB receptor thereby activating downstream molecules that can promote neurogenesis and cell survival [105].

Ferulic acids attenuate the stress-induced behavior in the depression-like model in mice [101]. Ferulic acids influence the function of ionotropic receptors for GABA in the brain, therefore, enhancing its anxiolytic effects [92].

Terpenoids (such as monoterpenes, triterpenoid, and sesquiterpene alkaloid) isolated from the rhizome of *Valerian officinalis* exhibited a broad range of neuroprotective actions [106]. Terpenoids were shown to confer sedating effects in mice through the activation of GABA-A receptor activity and other pathway upstreams of nuclear factor erythroid 2-related factor 2 (Nrf2) [106].

Polyunsaturated fatty acids (PUFAs) such as oleic acid, linoleic acid, and *α*-linolenic acid maintain the integrity of the structural components of neurons [107]. The fatty acid composition of the neuronal membrane is necessary for the maintenance of appropriate electrical gradients across the membrane and neurotransmission in the synaptic cleft [108]. The PUFAs improve membrane fluidity and, therefore, affect membrane biophysical properties. In neuronal membranes, PUFAs participate in signaling cascades that promote synaptic plasticity, neuronal function, and neuroprotection [109].

## 5. Conclusion

Chronic consumption of a high-fat diet has been implicated in impairments of neurobehavioral domains such as locomotor activity, anxiety, and exploration. Increased central adiposity activates brains' resident immune cells the microglia and astrocytes to increase the release of proinflammatory cytokines such as TNF-*α*, interleukin (1*β* and 6), and monocytes and macrophages. A rise in the concentration of proinflammatory cytokines further increases generation of reactive oxygen species (ROS) such as hydroxyl (OH^**·**^), hydroperoxyl (OOH^−^), superoxide anion (O_2_^−^), alkoxyl (RO^−^), and peroxyl (ROO^−^) free radicals and reactive nitrogen species (RNS) such as nitric oxide (NO^−^), nitrogen dioxide (NO_2_), and peroxynitrite (ONOO^−^). The increased production of ROS and RNS precipitates an oxidative damage to the hippocampus. High levels of ROS and RNS also decrease dopamine synthesis, release, and striatal-dopamine receptor function thereby altering the motor faculties. The accumulation of the toxic amyloid-beta plaques due to facilitated central adiposity also disrupts the HPA axis and decreases the GABAergic tone and, therefore, results in anxious states.

The present study demonstrated that a higher frequency of cross lattice, rearing, and center square entries is indicative of increased locomotion and exploration-like behaviors. The high frequency of fecal, urination, and stretch attend postures score is indicative of anxiety. Besides, the increased freezing period, grooming patterns, and latency duration are suggestive of increased anxious states. The therapeutic application of graded doses of the *G. glauca* indicated anxiolytic effects and increased spontaneous locomotor activity and exploration-like behaviors in HFD-induced obese rats. The observed therapeutic effects might be attributed to the phytochemicals contained in the *G. glauca*. These phytocompounds have been shown to mitigate the main mediators of neurodegeneration, viz., inflammation and oxidative stress by downregulating the activity and release of proinflammatory mediators and restoration of redox homeostatic status through activation of antioxidant defenses. The findings of the present study provide “qualified leads” for the synthesis of new alternative antioxidant supplement and therapeutic agent for management of obesity and other associated symptomatic complications such as anxiety and impaired motor faculties. However, there is a need for further studies to establish the depth of this possibility.

## Figures and Tables

**Table 1:** High-fat diet composition.

Ingredients	Diet (g/kg)
Lard	290
Powdered NPDL	375
Casein	265
Vitamin and mineral mix	60
Cholesterol	10
Corn oil	10
DI methionine	03
Sodium chloride	01
Yeast powder	01

The ingredients and their respective quantities of the prepared high-fat diet.

**Table 2:** Effect of DCM leaf extract of *Gnidia glauca* on locomotor activities, anxiety, and exploration-like behaviors in HFD-induced obese rats.

Treatments (mg/kg bw)	Grid crossings	Rearing	Defecation score	Urination	Center square entries	Stretch attend postures	Latency period (sec)	Freezing (sec)	Grooming (sec)
Normal control	37.20 ± 1.92^c^	10.00 ± 1.58^b^	3.60 ± 0.55^b^	2.00 ± 0.71^b^	9.20 ± 1.48^a^	3.80 ± 0.84^b^	23.20 ± 2.39^b^	21.60 ± 2.70^b^	15.80 ± 2.77^b^
Negative control	21.80 ± 2.39^d^	3.20 ± 0.84^c^	9.00 ± 0.71^a^	5.60 ± 0.55^a^	3.20 ± 0.84^b^	11.00 ± 1.58^a^	38.80 ± 2.59^a^	46.20 ± 4.15^a^	29.00 ± 2.45^a^
Positive control	39.00 ± 2.24^c^	9.40 ± 1.14^b^	3.60 ± 0.55^b^	2.60 ± 0.55^b^	8.00 ± 1.58^a^	4.60 ± 1.14^b^	23.40 ± 2.30^b^	19.40 ± 2.41^bc^	13.80 ± 1.48^bc^
HFD+200	41.60 ± 2.97^bc^	11.20 ± 1.48^ab^	3.20 ± 0.84^b^	2.40 ± 0.89^b^	9.20 ± 1.79^a^	4.20 ± 1.30^b^	20.40 ± 1.14^bc^	17.80 ± 1.79^bcd^	12.40 ± 2.07^bc^
HFD+250	46.00 ± 4.30^ab^	11.00 ± 1.58^ab^	3.40 ± 0.55^b^	2.40 ± 0.55^b^	9.40 ± 1.14^a^	3.80 ± 0.84^b^	18.80 ± 1.92^cd^	15.60 ± 1.52^cd^	11.80 ± 1.30^c^
HFD+300	50.40 ± 3.36^a^	12.80 ± 1.30^a^	2.60 ± 0.55^b^	2.00 ± 1.00^b^	10.20 ± 1.30^a^	3.00 ± 0.71^b^	15.60 ± 2.30^d^	14.00 ± 1.58^d^	10.20 ± 1.30^c^

Results are expressed as mean ± SD. Means followed by similar lowercase letters within columns are not statistically different (*p* > 0.01). Analyzed by ANOVA followed by Tukey's post hoc test for multiple comparisons and separation of means among treatment groups.

**Table 3:** Quantity of phytochemical compounds in DCM leaf extract of *Gnidia glauca*.

RT	Compound name	Concentration (mg/kg)
15.19	Ferulic acid	10.18 ± 1.14
21.53	Flavonols	10.15 ± 1.58
23.06	Oleic acid	21.05 ± 2.34
24.73	3,5,-Dihydroxy-trans-stilbene (pinosylvin)	13.39 ± 4.06
24.92	Catechins	9.27 ± 2.05
25.44	Octadecanoic acid (stearic acid)	10.73 ± 1.55
26.35	9,12,15-Octadecatrienoic acid, (Z,Z,Z)-(*α*-linolenic acid)	9.74 ± 2.85
27.90	Eicosapentaenoic acid	7.62 ± 0.89
28.48	Docosahexaenoic acid	7.94 ± 0.44
29.22	Curcumin	16.91 ± 2.30
30.07	Phytol	11.04 ± 1.18
30.24	Quercetin	15.74 ± 1.01
30.79	*γ*-Sitosterol	18.84 ± 1.04
32.23	Gallocatechin-catechin flavan	10.40 ± 1.00
36.82	*α*-Amyrin	5.25 ± 0.78

Concentrations of compounds identified in *Gnidia glauca* leaf extract (mg/kg). Results are expressed as means ± SD for replicate measurement *n* = 3. RT is the retention time.

## Data Availability

No data was used to support this study.
